# Immunoscore Signatures in Surgical Specimens and Tumor-Infiltrating Lymphocytes in Pretreatment Biopsy Predict Treatment Efficacy and Survival in Esophageal Cancer

**DOI:** 10.1097/SLA.0000000000005104

**Published:** 2021-07-29

**Authors:** Toshiki Noma, Tomoki Makino, Kenji Ohshima, Keijiro Sugimura, Hiroshi Miyata, Keiichiro Honma, Kotaro Yamashita, Takuro Saito, Koji Tanaka, Kazuyoshi Yamamoto, Tsuyoshi Takahashi, Yukinori Kurokawa, Makoto Yamasaki, Kiyokazu Nakajima, Eiichi Morii, Hidetoshi Eguchi, Yuichiro Doki

**Affiliations:** * Department of Gastroenterological Surgery, Graduate School of Medicine, Osaka University, Suita City, Osaka, Japan; † Department of Pathology, Osaka University, Graduate School of Medicine, Suita City, Osaka, Japan; ‡ Department of Gastroenterological Surgery, Osaka International Cancer Institute, Osaka, Osaka, Japan; § Department of Pathology, Osaka International Cancer Institute, Osaka, Osaka, Japan

**Keywords:** biopsy, esophageal cancer, immunotherapy, neoadjuvant chemotherapy, survival, tumor-infiltrating lymphocytes

## Abstract

Tumor-infiltrating lymphocytes (TILs) have long been recognized as playing an important role in tumor immune microenvironment. Lately, the Immunoscore (IS) has been proposed as a new method of quantifying the number of TILs in association with patient survival in several cancer types.

**Methods:**

In 300 preoperatively untreated esophageal cancer (EC) patients who underwent curative resection at two different institutes, immunohistochemical staining using CD3 and CD8 antibodies was performed to evaluate IS, as objectively scored by auto-counted TILs in the tumor core and invasive margin. In addition, in pre-neoadjuvant chemotherapy (pre-NAC) endoscopic biopsies of a different cohort of 146 EC patients who received NAC, CD3, and CD8 were immunostained to evaluate TIL density.

**Results:**

In all cases, the IS-high (score 3–4) group tended to have better survival [5-year overall survival (OS) of the IS-high vs low group: 77.6 vs 65.8%, *P* = 0.0722] than the IS-low (score 1–2) group. This trend was more remarkable in cStage II–IV patients (70.2 vs 54.5%, *P* = 0.0208) and multivariate analysis of OS further identified IS (hazard ratio 2.07, *P* = 0.0043) to be an independent prognostic variable. In preNAC biopsies, NAC-responders had higher densities than non-responders of both CD3^+^ (*P* = 0.0106) and CD8^+^ cells (*P* = 0.0729) and, particularly CD3^+^ cell density was found to be an independent prognostic factor (hazard ratio 1.75, *P* = 0.0169).

**Conclusions:**

The IS signature in surgical specimens and TIL density in preNAC- biopsies could be predictive markers of clinical outcomes in EC patients.

Esophageal cancer (EC) is the eighth most common malignancy and the seventh leading cause of cancer death worldwide.^1^ Esophageal adenocarcinoma is predominant in Western countries, whereas esophageal squamous cell carcinoma accounts for the bulk of cancer incidence and mortality in Asian countries.^2^ Despite current development of multimodal treatments including surgery, radiotherapy, and chemotherapy, especially for advanced cases, EC patients still face a dismal prognosis.^3^ Also, although neoadjuvant chemotherapy (NAC) has become a standard treatment for locally advanced EC, survival benefit is limited to responders to NAC.^4^ Accordingly, to establish personalized medicine and improve survival in advanced EC patients, there is an urgent need for biomarkers that accurately predict patient survival or treatment efficacy.

In the recent ATTRACTION-3 Phase III trial, nivolumab, an anti-PD-1 antibody, was proven to significantly improve overall survival compared with a conventional taxan chemo-agent in patients with unresectable or recurrent esophageal cancer resistant to first- line chemotherapy.^5^ The recent development of immuno-checkpoint inhibitors (ICI) for several cancer types^6^ has highlighted the importance of the tumor immune microenvironment. It comprises many host cells, including cytotoxic or regulatory T cells, dendritic cells, macrophages (M1 and M2), B cells, myeloid-derived suppressor cells, among others.^7^ Among them, tumor-infiltrating lymphocytes (TILs), particularly cytotoxic T cells, are recognized as playing a central role in anti-tumor immunity, and the clinical application of adoptive immunotherapy using TILs has recently begun for some cancer types.^8^^–^ 11 In this regard, objective evaluation of TIL status in the tumor locus will be extremely important in establishing personalized treatments, including immunotherapy for EC. Recently, the Immunoscore (IS), which quantifies the number of TILs (CD3^+^ and CD8^+^ lymphocytes) in the core of the tumor (CT) and invasive margin (IM), has been proposed as a new method of TIL assessment, and a higher number of TILs (ie, a high IS score), has been reported to be associated with better prognosis in several types of cancer.^12^^–^ 22 However, in EC there has been no evidence of the feasibility and clinical utility of the IS in predicting patient prognosis.^23^ Therefore, this study aimed to establish and standardize a TIL assessment in surgical specimens and pretreatment endoscopic biopsies of EC to evaluate their utility in predicting prognosis and therapeutic effect.

## Material and Methods

### Patients

A total of 300 consecutive patients with preoperatively untreated esophageal squamous cell carcinoma who underwent curative esophagectomy at 2 different institutes, Osaka University Hospital (n = 162) and Osaka International Cancer Institute (n = 138), between March 2000 and September 2017 were enrolled in the study. All formalin-fixed paraffin-embedded (FFPE) tissues containing the deepest part of the tumor obtained from the two institutions were used for immunohistochemical (IHC) staining and analysis of IS. To further evaluate TILs in pre-NAC endoscopic biopsy samples, a different cohort of 146 EC patients who underwent surgical resection after NAC (DCF: socetaxel, cisplatin, and 5- fluorouracil; or FAP: 5-fluorouracil, adriamycin, and cisplatin) at Osaka University Hospital^24^^–^ 30 were also analyzed. Cases with other cancer types and multiple cancers were excluded. The patients’ clinical and pathological data were obtained through medical charts and pathology reports. Information on patient outcomes and survival data were collected. Tumor stage was classified according to the 8th edition of the UICC/AJCC (Union for International Cancer Control/ American Joint Committee on Cancer) TNM classification system.^31^ This study was performed with the approval of the Ethics Committee of Osaka University Hospital and Osaka International Cancer Institute, and informed consent was obtained from all participants.

### Immunohistochemistry of CD3 and CD8

A pathologist (K.O.), who was unaware of the clinical data, selected all the FFPE tissues containing the deepest part of the tumor and invasive margin. The distribution and density of CD3^+^ and CD8^+^ lymphocytes in surgical specimens of primary EC were evaluated using IHC with affinity-purified mouse monoclonal antibodies against CD3 (Clone F7.2.38, Dako, 1:250 dilution) and CD8 (Clone C8.144B, Dako, 1:500 dilution). The specificities of these monoclonal antibodies in IHC on paraffin-embedded samples were confirmed with human tonsil tissue sections (positive control). All FFPE tissues were cut into 4-µm sections, deparaffinized in xylene, and rehydrated through an ethanol gradient. For antigen retrieval, the sections were boiled for 20 minutes in a pressure cooker at 110°C in antigen-retrieval buffer (pH 6.0). Slides were peroxidase-blocked in 0.3% H_2_O_2_ in methanol for 20 min, then blocked with normal horse serum (S-2000, Vector Laboratories) at room temperature for 20 min in humid boxes and then incubated at 4°C overnight with mouse monoclonal anti-CD3 or anti-CD8. Next, they were washed with 1% PBS, then incubated with secondary antibody (S-2000 and BA-2000, Vector) at room temperature for 20 minutes. The slides were then washed with PBS. The biotinylated secondary antibodies were reacted against by using Avidin-Biotin Complex Staining Kits (Vectastain ABC Kit, PK6100, Vector) at room temperature for 20 min. These slides were again washed with PBS, and the staining was visualized by incubation with DAB (Wako) for about 2.5 minutes. The sections were counterstained with hematoxylin, dehydrated in ethanol, cleared in xylene, and coverslipped.^32,33^

### Evaluation of the IS in Resected Specimens and TILs in Pretherapeutic Endoscopic Biopsies

**Figure 1:**
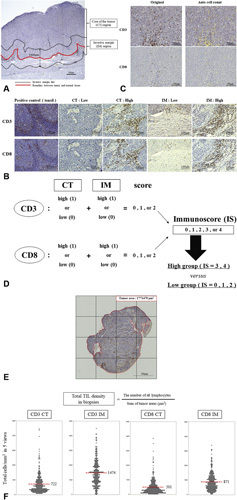
Immunostaining and automated counts of CD3^+^ and CD8^+^ cells for Immunoscore evaluation. A, Representative CD3 immunostaining section in an EC resected specimen indicating typical tumor regions core of tumor (CT) and invasive margin (IM) (original magnification: 20×). B, Positive control (tonsil) and representative slides of the low or high density of CD3^+^ and CD8^+^ lymphocytes in the CT or IM, respectively. C, Auto count of the density of CD3^+^ and CD8^+^ lymphocytes by using the image analysis system (BZ-X710 digital microscope analyzer, Keyence, Osaka, Japan). Yellow dots indicate immunostained area. D, Schematic of the Immunoscore (IS) model. E, Representative slide of CD3 immunostaining of a pre-therapeutic biopsy and auto-count of the CD3^+^ lymphocyte number. This number was added to the similarly acquired CD8^+^ lymphocyte number and the sum was divided by the total biopsy tumor area of a single section (the area inside the red line) to calculate the total TIL density. F, Scatter dot plots of total cell densities (cells/mm^2^) of the top 5 views counted CD3^+^ and CD8^+^ lymphocytes in the CTor IM. Red dotted lines represent the respective average values.

The IS is a quantification system based on the combination of 2 markers (CD3 and CD8) in 2 regions (the CT and IM).^15^^–^ 17 The IM region is defined as the area 500 µm inward and outward from the boundary between normal tissue and tumor tissue, and the CT region is defined as all tumor areas interior to the IM region (Fig. 1A).^23,34^ Multiple tiles (from 1-tiled view of 500 × 500 µm) with large numbers of stained TILs were selected in both the CT and IM of each primary tumor surgical specimen.

The top 5 TIL “hotspots” (5 tiles with largest numbers of TILs) were selected for TIL counting using IHC in both the center and invasive margin of the tumor surgical specimen (Fig. 1B).^17^ We counted the number of CD3^+^ and CD8^+^ lymphocytes automatically, using a BZ-X710 digital microscope at 200× magnification (Keyence, Osaka, Japan) and hybrid cell-counting software (BZ-H3C; Keyence) (Fig. 1C). The number of TILs was scored (0–2 points) by using a cutoff value of mean density in 5 hotspots (CD3 and CD8, respectively). Finally, the sum of each score was used to classify the tumors into two groups [the IS-low (0–2 points) vs the IS-high (3–4 points) group] and the correlation between the IS model and clinico-pathological variables including survival was evaluated (Fig. 1D).^13^,^15^ All slides were assessed independently by 2 observers (T.N. and T.M.) blinded to the clinico-pathological data and then by conference in case of disagreement. One pathologist (K.O.) confirmed the final diagnosis.^35^ To evaluate TILs in pretreatment biopsies, biopsy samples were immunostained separately with CD3 and CD8 antibodies using the method described above. The total number of all lymphocytes was autocounted and divided by the sum of all biopsy tumor areas as the total TIL density (Fig. 1E),^36^ and the median value was used as the cutoff value to categorize tumors to the two groups. The correlation between TIL density and clinicopathological variables including response to NAC and patient prognosis was evaluated.

### Statistical Analysis

Continuous variables were expressed as means and standard deviations, and means were compared using the *t* test. The survivaltime distribution was evaluated using the Kaplan-Meier method. To evaluate independent prognostic significance and relative risk, we performed univariate analysis of clinicopathological factors. Any variables that were significant in the univariate analyses were included in multivariate analyses. Cox logistic regression was used to perform the multivariate analyses. We considered a *P* value <0.05 to be statistically significant. All statistical calculations were performed using JMP version 14 software (SAS Institute, Cary, NC).

## Results

### Relationship Between the IS and Patients’ Clinicopathological Variables

The numbers of cells per area of TILs (total cells/mm^2^ in the top 5 views) in the CT and IM regions as immunostained using CD3 and CD8 antibodies are shown in Figure 1F. The average numbers of CD3^+^ lymphocytes were 722/mm^2^ in the CT and 1474/mm^2^ in the IM (*P* = 0.0001). The average numbers of CD8^+^ lymphocytes were 500/mm^2^ in the CT and 870/mm^2^ in the IM (*P* = 0.0001). Accordingly, the distribution of the IS was 0 (n = 92), 1 (n = 76), 2 (n = 52), 3 (n = 32), and 4 (n = 48). When all patients were divided into 2 groups (IS-high vs IS-low), there was no statistically significant association between the IS and clinico-pathological variables including age, sex, tumor location, histological differentiation, pT, pN, pM, pStage, lymphatic invasion, or vascular invasion, as shown in Table 1.

**Table 1:** Correlation Between Clinicopathological Variables and Immunoscore in All Patients With No Preoperative Treatment (n = 300)

	IS–High Group (n = 80, %)	IS–Low Group (n = 220, %)	*P*
Age			0.2401
Median (range)	69 (43–85)	66 (44–85)	
Sex			
Male	67 (83.7%)	178 (80.9%)	0.5700
Female	13 (16.3%)	42 (19.1%)	
Tumor location			0.1791
Ut	9 (11.2%)	40 (18.2%)	
Mt	42 (52.5%)	120 (54.5%)	
Lt	29 (36.3%)	60 (27.3%)	
Histological differentiation (SCC)			0.8286
Well	16 (20.0%)	42 (19.1%)	
Mod	51 (63.7%)	150 (68.2%)	
Poor	11 (13.8%)	25 (11.4%)	
Others	2 (2.5%)	3 (1.3%)	
pT			0.0853
1	36 (45.0%)	113 (51.4%)	
2	7 (8.7%)	30 (13.6%)	
3	36 (45.0%)	68 (30.9%)	
4	1 (1.3%)	9 (4.1%)	
pN			0.4281
0	36 (45.0%)	118 (53.6%)	
1	28 (35.0%)	56 (25.4%)	
2	11 (13.7%)	33 (15.0%)	
3	5 (6.3%)	13 (5.9%)	
pM			0.1234
0	74 (92.5%)	213 (96.8%)	
1	6 (7.5%)	7 (3.2%)	
pStage			0.2700
I	26 (32.5%)	94 (42.7%)	
II	25 (31.2%)	57 (25.9%)	
III	23 (28.8%)	61 (27.7%)	
IV	6 (7.5%)	8 (3.7%)	
Lymphatic invasion			0.5887
0	29 (36.3%)	93 (42.3%)	
1	30 (37.5%)	85 (38.6%)	
2	17 (21.2%)	34 (15.5%)	
3	4 (5.0%)	8 (3.6%)	
Vascular invasion			0.7301
0	36 (45.0%)	115 (52.3%)	
1	34 (42.5%)	81 (36.8%)	
2	8 (10.0%)	20 (9.1%)	
3	2 (2.5%)	4 (1.8%)	

### Prognostic Impact of IS on EC Patients With No Preoperative Treatment

**Figure 2:**
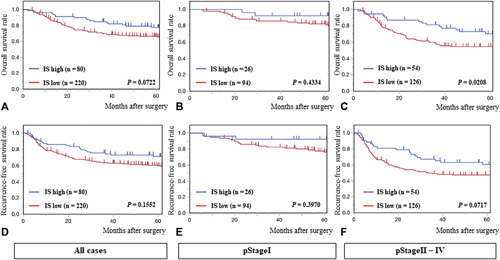
Kaplan-Meier survival curves of overall survival (OS) in (A) all EC cases, (B) pStage I tumors, and (C) pStage II-IV tumors; and recurrence-free survival (RFS) in (D) all cases, (E) pStage I tumors, and (F) pStage II-IV tumors according to the IS.

In all EC cases without any preoperative treatments, the IS-high group tended to have better overall survival (OS) (5-year OS of IS-high vs low group; 77.6% vs 65.8%, respectively, *P* = 0.0722) and recurrence-free survival (RFS) (5-year RFS of IS-high vs IS-low group; 71.3% vs 59.7%, respectively, *P* = 0.1552) compared with the IS-low group, but the differences were not statistically significant (Fig. 2A, D). In stage I tumors, there was no significant difference in OS (*P* = 0.4334) or RFS (*P* = 0.3970) between the IS-high and IS-low groups, as shown in Figure 2B and E. However, in patients with stage II-IV tumors (n = 180), the IS-high group was significantly associated with better OS than the IS-low group (5-year OS of the IS-high vs low group; 70.2% vs 54.5%, respectively, P = 0.0208) and RFS (5-year RFS of the IS-high vs low group; 60.6% vs 47.3%, respectively, *P* = 0.0717) (Fig. 2C, F). In univariate analysis of OS, differences in histological differentiation, pN, pM, and lymphatic invasion were found to be statistically significant prognostic factors (Table 2). Multivariate analysis further identified pN [hazard ratio (HR) 1.84, 95% confidence interval (CI) 1.16–2.92, *P* = 0.0093], pM (HR 2.70,95%CI1.36–5.38, *P* = 0.0047), and IS (HR 2.07, 95% CI 1.26–3.41, *P* = 0.0043) to be independent prognostic factors, as shown in Table 2

**Table 2:** Uni-and Multivariate Analysis of Overall Survival in pStageII-IV Patients With No Preoperative Treatment (n = 180)

	Univariate Analysis		Multivariate Analysis	
	HR (95% CI)	*P*	HR (95% CI)	*P*
Age, y		0.0870		
≥70	1.43 (0.95–2.18)			
<70	1			
Sex		0.8929		
Male	1.04 (0.59–1.84)			
Female	1			
Location		0.2588		
Ut	1			
Mt/Lt	1.35 (0.80–2.26)			
Histological differentiation (SCC)		**0.0119**		0.1120
Well/mod	1		1	
Poor/basoloid	1.90 (1.15–3.12)		1.51 (0.90–2.51)	
pT		0.9734		
1, 2	1			
3, 4	1.01 (0.65–1.56)			
pN		**0.0011**		**0.0093**
0, 1	1		1	
2, 3	2.12 (1.35–3.33)		1.84 (1.16–2.92)	
pM		**0.0013**		**0.0047**
0	1		1	
1	2.98 (1.53–5.78)		2.70 (1.36–5.38)	
Lymphatic invasion		**0.0146**		0.0773
0	1		1	
1, 2, 3	2.03 (1.15–3.40)		1.70 (0.94–3.07)	
Vascular invasion		0.9229		
0	1			
1, 2, 3	0.98 (0.64–1.50)			
Immunoscore		**0.0226**		**0.0043**
Low: 0, 1, 2	1.77 (1.08–2.88)		2.07 (1.26–3.41)	
High: 3, 4	1		1	

### TIL Evaluation in Pretherapeutic Endoscopic Biopsies in EC Patients

**Figure 3:**
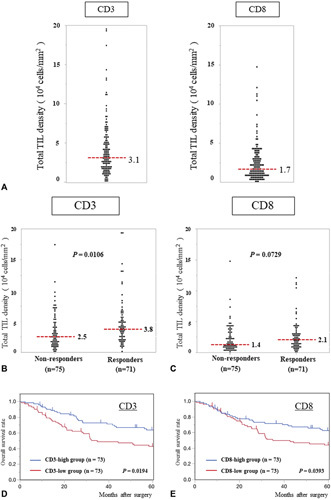
A, Scatter dot plots of total densities of CD3^+^ and CD8^+^ TILs;the red dotted lines represent median values. B and C, Scatter dot plots of the total densities of (B) CD3^+^ and (C) CD8^+^ TILs according to histological response to NAC. Red dotted lines represent the median values. D and E, Kaplan-Meier survival curves for overall survival (OS) according to the total densities of (D) CD3^+^ and (E) CD8^+^ TILs.

We next evaluated the total TIL density in pretherapeutic endoscopic biopsies, using IHC with CD3 and CD8 antibodies in a different cohort of 146 EC patients with NAC, as shown in Figure 3A. The median densities of CD3^+^ and CD8^+^ cells were 3.1 × 10^4^/µm^2^ and 1.7 × 10^4^/µm^2^, respectively (*P* = 0.0001). There was no statistically significant difference between any clinico-pathological variable and total TIL density (CD3^+^ or CD8^+^), although high CD8^+^ cell density tended to be associated with poor differentiation of squamous cell carcinoma (*P* = 0.0822) and vascular invasion (*P* = 0.0581), as shown in Table 3.

**Table 3:** Correlation Between Clinicopathological Parameters and TILs (CD3^+^ and CD8^+^) in a Pretherapeutic Tumor Biopsy Cohort (N = 146)

	CD3			CD8		
	High Group (n = 73)	Low Group (n = 73)	*P*	High Group (n = 73)	Low Group (n = 73)	*P*
Age, y			0.3093			0.1104
Median (range)	67 (36–79)	66 (38–83)		67 (36–79)	64 (38–83)	
Sex			0.2544			0.2544
Male	59 (80.8%)	64 (87.7%)		59 (80.8%)	64 (87.7%)	
Female	14 (19.2%)	9 (12.3%)		14 (19.2%)	9 (12.3%)	
Location			0.4503			1.0000
Ut	21 (28.8%)	17 (23.3%)		19 (26.0%)	19 (26.0%)	
Mt/Lt	52 (71.2%)	56 (76.7%)		54 (74.0%)	54 (74.0%)	
Histological differentiation (SCC)			0.1151			0.0822
Well/mod	55 (87.3%)	53 (76.8%)		57 (87.7%)	51 (76.1%)	
Poor	8 (12.7%)	16 (23.2%)		8 (12.3%)	16 (23.9%)	
NAC regimen			0.7406			1.0000
DCF	38 (52.1%)	36 (49.3%)		37 (50.7%)	37 (50.7%)	
FAP	35 (47.9%)	37 (50.7%)		36 (49.3%)	36 (49.3%)	
cT			0.1736			0.3318
1, 2	14 (19.2%)	52 (71.2%)		58 (79.4%)	53 (72.6%)	
3, 4	59 (80.8%)	21 (28.8%)		15 (20.6%)	20 (27.4%)	
cN			0.1895			0.8516
0, 1	23 (31.5%)	16 (21.9%)		20 (27.4%)	19 (26.0%)	
2, 3	50 (68.5%)	57 (78.1%)		53 (72.6%)	54 (74.0%)	
cM			0.5958			1.0000
0	9 (12.3%)	7 (9.6%)		8 (11.0%)	8 (11.0%)	
1	64 (87.7%)	66 (90.4%)		65 (89.0%)	65 (89.0%)	
cStage			0.7272			1.0000
I, II	24 (32.9%)	26 (83.7%)		25 (34.2%)	25 (34.2%)	
III, IV	49 (67.1%)	47 (16.3%)		48 (65.8%)	48 (65.8%)	

DCF indicates docetaxel/cisplatin/5–fluorouracil; FAP, 5–fluorouracil/adriamycin/cisplatin.

### TIL Density in Pretherapeutic Biopsies Predicts Response to Neoadjuvant Chemotherapy and Long-Term Survival

The correlation between the total CD3^+^ and CD8^+^ TIL density in pre-NAC endoscopic biopsies and pathological response to NAC was analyzed as shown in Figure 3B and C. Compared with NAC responders (pathological response: grade 2–3), nonresponders (grade 0–1b) had significantly lower CD3^+^ cell density (3.8 × 10^4^/µm^2^ vs 2.5 × 10^4^/µm^2^, respectively, *P* = 0.0106). Similarly, CD8^+^ cell density tended to be larger in responders compared with non–responders (2.1 × 10^4^/µm^2^ vs 1.4 × 10^4^/µm^2^, respectively, *P* = 0.0729). Univariate analyses of factors predicting pathological response showed that NAC regimen (*P* = 0.0210), and CD3^+^ (*P* = 0.0053) and CD8^+^ (*P* = 0.0696) cell density in pretreatment biopsies were statistically significant. Multivariate analysis further identified both CD3^+^ [odds ratio (OR) 1.75, 95% CI 1.12–2.78, *P* = 0.0169] and CD8^+^ cell density (OR 1.88, 95% CI 0.96–3.68, *P* = 0.0169) as independent predictors of NAC efficacy, in addition to NAC regimen (Supplemental Table 1, http://links.lww.com/SLA/D310). In terms of survival analysis, the CD3^+^-high group showed significantly better OS than the CD3^+^-low group (5-year OS of CD3^+^-high vs CD3^+^-low group: 63.9% vs 42.9%, *P* = 0.0194) (Fig. 3D). Similarly, the CD8^+^-high group was associated with the better survival compared with the CD8^+^-low group (5-year OS of CD8^+^-high vs CD8^+^-low group: 62.7% vs 44.2%, *P* = 0.0393) (Fig. 3E). In uni-and multivariate analyses of OS, CD3^+^ cell density in pre–therapeutic endoscopic biopsies (HR 1.75, 95% CI 1.12–2.78, *P* = 0.0169) and cM (HR 2.15, 95% CI 1.16–4.00, *P* = 0.0156) were identified as independent prognostic factors, whereas the CD8^+^ cell density was not (HR 1.57, 95% CI 0.99–2.49, *P* = 0.0561) (Table 4).

**Table 4:** Uni- and Multivariate Analysis of Overall Survival in Pretherapeutic Tumor Biopsy Cohort (N = 146)

	Univariate Analysis		Multivariate Analysis			
			Model A		Model B	
	HR (95% CI)	*P*	HR (95% CI)	*P*	HR (95% CI)	*P* value
Age, y		0.2904				
>70	1.30 (0.80–2.13)					
<70	1					
Sex		0.8351				
Male	1.07 (0.56–2.03)					
Female	1					
Location		0.7264				
Ut	1					
Mt/Lt	1.09 (0.65–1.85)					
Histological differentiation (SCC)		0.9194				
Well/mod	1					
Poor	0.97 (0.53–1.78)					
NAC-regimen		0.7147				
DCF	1					
FAP	1.10 (0.69–1.72)					
cT		0.4793				
1, 2	1					
3,4	1.22 (0.70–2.12)					
cN		0.9443				
0, 1	1					
2, 3	1.02 (0.61–1.70)					
cM		**0.0206**		**0.0156**		**0.0306**
0	1		1		1	
1	2.08 (1.12–3.86)		2.15 (1.16–4.00)		1.98 (1.07–3.69)	
CD3^+^ density		**0.0209**		**0.0169**		
Low: 0, 1, 2	1.72 (1.09–2.73)		1.75 (1.12–2.78)			
High: 3, 4	1		1			
CD8^+^ density		**0.0412**				0.0561
Low: 0, 1, 2	1.62 (1.02–2.56)				1.57 (0.99–2.49)	
High: 3, 4	1				1	

DCF indicates docetaxel/cisplatin/5–fluorouracil; FAP, 5–fluorouracil/adriamycin/cisplatin.

## Discussion

In this study we evaluated the prognostic value of the IS as objectively scored using automated cell counts performed with a digital microscope and hybrid cell-counting software. Using resected specimens of a large series (n = 300) of preoperatively untreated EC patients from two institutes, we found a significant correlation between IS and patient prognosis, especially in pStage II–IV cases. The IS was identified as an independent prognostic factor by multivariate analysis of OS in advanced EC cases. We further evaluated TILs in pretherapeutic endoscopic biopsies, and showed that CD3^+^ or CD8^+^ cell density was significantly associated with pathological response to NAC. In addition, CD3^+^ cell density in endoscopic biopsies was found to be an independent prognostic factor, indicating the clinical utility of evaluating TILs in both resected specimens and endoscopic biopsies to predict treatment outcomes in EC patients.

In several cancer types, including malignant melanoma, breast, and colorectal cancer, evidence has already established TIL density as an immuno–oncological biomarker, and proposals have been made to include TILs among the markers used for routine histopathological diagnosis in clinical practice.^16,23,37–39^ Here, by applying the IS model to EC, we also explored using TILs as possible prognostic biomarkers. The decision to use CD3 and CD8 antibodies in evaluating the IS model was originally based on the possibility that hematoxylin-eosin staining alone would be insufficient to quantify TILs, and on the high quality of the staining and stability of the antibodies to the antigens selected as IS markers. We found a significant correlation between IS and prognosis only in advanced cases, in agreement with a previous report regarding gastric cancer by Jiang et al.^17^ These results might be explained by the difficulty in evaluating TILs only by hotspots in early–stage cases with much smaller tumor volumes than advanced cases. In addition, early–stage EC cases have many tertiary lymphoid structures, which are classically defined as lymphoid aggregates forming in nonhematopoietic organs in response to chronic and nonresolving inflammatory processes. They may cause the immune response to the tumor in the mucosal lamina propria to differ from that in advanced cases.^40^^–^ 44 Therefore, in early–stage cases, it may be difficult to discover an association with prognosis by simply counting TILs.

In our TIL evaluation of resected specimens, the density of CD3^+^ cells was naturally higher than that of CD8^+^ cells, and significantly higher in the IM region than in the CT. In this regard, Wang et al reported similar results regarding colorectal cancer (and liver metastasis), whereas Li et al reported lower CD3^+^ cell density in the IM than in the CT in bladder cancer.^18,45,46^ This discrepancy of TIL count across cancer types even when the same IS method is used may be partly due to differences in tumor stromal volume among different cancers. Although we have not evaluated stromal and tumor areas separately in this study, we speculate that squamous cell carcinoma has a tissue structure that makes it difficult for lymphocytes to infiltrate the tumor because of the relatively small stromal volume inside the tumor. As a result, the number of lymphocytes may be higher in the IM than in the CT.^35^ In this study, the TIL count in the CT correlated better with prognosis than did the count in the IM (data not shown), which may indicate that the lymphocytes infiltrating the CT have stronger antitumor activity. In addition, the prognosis was better stratified by the IS combining both CD3^+^ and CD8^+^ cells than by that using either CD3^+^ or CD8^+^ cells alone, whereas, as a single marker, CD3^+^ cells alone correlated better with prognosis than did CD8^+^ cells (data not shown). This result is in agreement with the previous report of IS evaluation in colorectal cancer by Galon et al.^47^ We considered the possibility that, among total TILs, immunocytes other than CD8^+^ cells, such as CD4^+^ and CD45RO^+^ cells, may also play an important role in anti-tumor immunity. In fact, the presence of CD45RO^+^ cells has been reported to be an independent prognostic factor in EC.^48^

In several cancer types, including EC, no standardized methods exist for evaluating TILs in biopsy samples by IHC.^49^^–^ 53 Earlier, we actually failed to evaluate IS in post-NAC resected specimens of EC because the residual cancer cells are usually scattered, creating islands in the stroma, especially in resected specimens of NAC responders, thus making it almost impossible to distinguish between the CT and IM.^15^ A potential solution is to assess TILs in pre-NAC endoscopic biopsies.^54^ In this study, endoscopic biopsy samples were evaluated by summing the total number of TILs in multiple biopsies to calculate their density, because tumor volumes in endoscopic biopsy samples are much smaller than they are in resected specimens and are therefore easily affected by tissue heterogeneity across tumor sampling sites. As the result, the high density of either CD3^+^ or CD8^+^ cells in pre–NAC biopsies was associated with favorable prognosis and better NAC response, and this trend was more prominent with CD3^+^ cells, as observed in the TIL evaluation of resected specimens (ie, the IS). These results indicate that TIL assessment using pre-NAC endoscopic biopsies has the potential to predict the therapeutic effect of NAC and long-term survival. This would be a clinical advantage and could lead to personalized medicine for EC patients: if a tumor were predicted to be a non-responder to NAC, other treatments than NAC, including ICI, would be chosen as first–line therapy.^55,56^

This study has several limitations. First, it is a retrospective analysis lacking independent sample validation. Second, the IS evaluation in this study was based on only 1 slide per tumor of the largest and deepest area in the specimen, rather than multiple or all slides. Third, tumor immunological factors, including PD-L1/2 expression, were not evaluated.^6,19,57^ In fact, Teng et al reported that so-called “hot tumors” with both high expression of PD-L1 and high TIL numbers in malignant melanoma are the most responsive tumors to ICI.^58^^–^ 61 Accordingly, it is important to establish a new evaluation criterion that combines TIL density and other immune-related factors to further improve prognostic accuracy.

## Conclusions

This study is the first to examine the IS, a new method of TIL assessment using IHC and automated cell count–based scoring, in resected specimens of EC patients, and found its significant association with long-term survival, especially in advanced cases. In addition, TIL density in pretherapeutic endoscopic biopsies was also shown to be useful in predicting both response to NAC and prognosis. The present results may contribute to the establishment of personalized medicine based on TIL evaluation in EC samples, which ultimately could improve survival in EC patients.

## Supplementary Material

## References

[1] Fitzmaurice C Abate D Abbasi N . Global, regional, and national cancer incidence, mortality, years of life lost, years lived with disability, and disability–adjusted life-years for 29 cancer groups, 1990 to 2017: a systematic analysis for the global burden of disease study. JAMA Oncol. 2019;5:1749–1768. 31560378 10.1001/jamaoncol.2019.2996 PMC6777271

[2] Chen S Zhou K Yang L . Racial differences in esophageal squamous cell carcinoma: incidence and molecular features. Biomed Res Int. 2017;2017:1204082. 28393072 10.1155/2017/1204082 PMC5368356

[3] Shah MA Kennedy EB Catenacci DV . Treatment of locally advanced esophageal carcinoma: ASCO guideline. J Clin Oncol. 2020;38:2677–2694. 32568633 10.1200/JCO.20.00866

[4] Ando N Kato H Igaki H . A randomized trial comparing postoperative adjuvant chemotherapy with cisplatin and 5–fluorouracil versus preoperative chemotherapy for localized advanced squamous cell carcinoma of the thoracic esophagus (JCOG9907). Ann Surg Oncol. 2012;19:68–74. 21879261 10.1245/s10434-011-2049-9

[5] Kato K Cho BC Takahashi M . Nivolumab versus chemotherapy in patients with advanced oesophageal squamous cell carcinoma refractory or intolerant to previous chemotherapy (ATTRACTION-3): a multicentre, randomised, openlabel, phase 3 trial. Lancet Oncol. 2019;20:1506–1517. 31582355 10.1016/S1470-2045(19)30626-6

[6] Hinshaw DC Shevde LA . The tumor microenvironment innately modulates cancer progression. Cancer Res. 2019;79:4557–4567. 31350295 10.1158/0008-5472.CAN-18-3962 PMC6744958

[7] Joyce JA Fearon DT . T cell exclusion, immune privilege, and the tumor microenvironment. Science. 2015;348:74–80. 25838376 10.1126/science.aaa6204

[8] Restifo NP Dudley ME Rosenberg SA . Adoptive immunotherapy for cancer: harnessing the T cell response. Nat Rev Immunol. 2012;12:269–281. 22437939 10.1038/nri3191 PMC6292222

[9] Besser MJ Shapira-Frommer R Treves AJ . Clinical responses in a phase II study using adoptive transfer of short-term cultured tumor infiltration lymphocytes in metastatic melanoma patients. Clin Cancer Res. 2010;16:2646–2655. 20406835 10.1158/1078-0432.CCR-10-0041

[10] Tran E Robbins PF Lu Y-C . T-cell transfer therapy targeting mutant KRAS in cancer. N Engl J Med. 2016;375:2255–2262. 27959684 10.1056/NEJMoa1609279 PMC5178827

[11] Jiang X Xu J Liu M . Adoptive CD8+ T cell therapy against cancer: challenges and opportunities. Cancer Lett. 2019;462:23–32. 31356845 10.1016/j.canlet.2019.07.017

[12] Galon J Pagès F Marincola FM . The immune score as a new possible approach for the classification of cancer. J Transl Med. 2012;10:1–4. Published online 2012;2–5. 22214470 10.1186/1479-5876-10-1 PMC3269368

[13] Anitei MG Zeitoun G Mlecnik B . Prognostic and predictive values of the immunoscore in patients with rectal cancer. Clin Cancer Res. 2014;20:1891–1899. 24691640 10.1158/1078-0432.CCR-13-2830

[14] Wu ZY Shen W Yue JQ . Combining immunoscore with clinicopatho– logic features in cholangiocarcinoma: an influential prognostic nomogram. Onco Targets Ther. 2020;13:11359–11376. 33192071 10.2147/OTT.S274754 PMC7654544

[15] Galon J Mlecnik B Bindea G . Towards the introduction of the “Immunoscore” in the classification of malignant tumours. J Pathol. 2014;232:199–209. 24122236 10.1002/path.4287 PMC4255306

[16] Pagès F Mlecnik B Marliot F . International validation of the consensus Immunoscore for the classification of colon cancer: a prognostic and accuracy study. Lancet. 2018;391:2128–2139. 29754777 10.1016/S0140-6736(18)30789-X

[17] Jiang Y Zhang Q Hu Y . ImmunoScore signature: a prognostic and predictive tool in gastric cancer. Ann Surg. 2018;267:504–513. 28002059 10.1097/SLA.0000000000002116

[18] Li XD Huang CW Liu ZF . Prognostic role of the immunoscore for patients with urothelial carcinoma of the bladder who underwent radical cystectomy. Ann Surg Oncol. 2019;26:4148–4156. 31376036 10.1245/s10434-019-07529-y

[19] Bruni D Angell HK Galon J . The immune contexture and Immunoscore in cancer prognosis and therapeutic efficacy. Nat Rev Cancer. 2020;20:662–680. 32753728 10.1038/s41568-020-0285-7

[20] Angell HK Bruni D Carl Barrett J . The immunoscore: Colon cancer and beyond a C. Clin Cancer Res. 2020;26:332–339. 31413009 10.1158/1078-0432.CCR-18-1851

[21] Zhang X Yang J Du L . The prognostic value of Immunoscore in patients with cancer: a pooled analysis of 10,328 patients. Int J Biol Markers. 2020;35:3–13. 10.1177/1724600820927409 32538254

[22] Nie RC Yuan SQ Wang Y . Robust immunoscore model to predict the response to anti–PD1 therapy in melanoma. Aging (Albany NY). 2019;11:11576–11590. 31796647 10.18632/aging.102556 PMC6932919

[23] Hendry S Salgado R Gevaert T . Assessing tumor–infiltrating lymphocytes in solid tumors. Adv Anat Pathol. 2017;24:235–251. 28777142 10.1097/PAP.0000000000000162 PMC5564448

[24] Sugase T Makino T Yamasaki M . Histological changes of superficial esophageal squamous cell carcinoma after preoperative chemotherapy. Esophagus. 2018;15:263–271. 10.1007/s10388-018-0626-8 29909488

[25] Hagi T Makino T Yamasaki M . Pathological regression of lymph nodes better predicts long-term survival in esophageal cancer patients undergoing neoadjuvant chemotherapy followed by surgery. Ann Surg. 2022;275:1121–1129. 32910622 10.1097/SLA.0000000000004238 PMC10060043

[26] Hashimoto T Makino T Yamasaki M . The pattern of residual tumor after neoadjuvant chemotherapy for locally advanced esophageal cancer and its clinical significance. Ann Surg. 2020;271:875–884. 30829694 10.1097/SLA.0000000000003129

[27] Urakawa S Makino T Yamasaki M . Lymph node response to neoadjuvant chemotherapy as an independent prognostic factor in metastatic esophageal cancer. Ann Surg. 2021;273:1141–1149. 31274656 10.1097/SLA.0000000000003445

[28] Makino T Yamasaki M Tanaka K . Metabolic tumor volume change predicts long–term survival and histological response to preoperative chemotherapy in locally advanced esophageal cancer. Ann Surg. 2019;270:1090–1095. 29727327 10.1097/SLA.0000000000002808

[29] Shiraishi O Makino T Yamasaki M . Two versus three courses of preoperative cisplatin and fluorouracil plus docetaxel for treating locally advanced esophageal cancer: short–term outcomes of a multicenter randomized phase II trial. Esophagus. 2021;18:825–834. 33738656 10.1007/s10388-021-00831-3

[30] Yamamoto K Makino T Sato E . Tumor–infiltrating M2 macrophage in pretreatment biopsy sample predicts response to chemotherapy and survival in esophageal cancer. Cancer Sci. 2020;111:1103–1112. 31981293 10.1111/cas.14328 PMC7156837

[31] Paul FT . Breast tumours. J R Soc Med. 1914;7(Surg_Sect):276–279. 10.1177/003591571400702073 PMC2003100 19978435

[32] Makino T Yamasaki M Takemasa I . Dickkopf–1 expression as a marker for predicting clinical outcome in esophageal squamous cell carcinoma. Ann Surg Oncol. 2009;16:2058–2064. 19408050 10.1245/s10434-009-0476-7

[33] Makino T Yamasaki M Takeno A . Cytokeratins 18 and 8 are poor prognostic markers in patients with squamous cell carcinoma of the oesophagus. Br J Cancer. 2009;101:1298–1306. 19755983 10.1038/sj.bjc.6605313 PMC2768453

[34] Galon J Pagès F Marincola FM . Cancer classification using the Immunoscore: a worldwide task force. J Transl Med. 2012;10:205. 23034130 10.1186/1479-5876-10-205 PMC3554496

[35] Society JE . Japanese Classification of Esophageal Cancer, 11th Edition: part II and III. Esophagus. 2017;14:37–65. 28111536 10.1007/s10388-016-0556-2 PMC5222925

[36] Lim YJ Koh J Kim S . Chemoradiation–induced alteration of programmed death-ligand 1 and CD8+ tumor-infiltrating lymphocytes identified patients with poor prognosis in rectal cancer: a matched comparison analysis. Int J Radiat Oncol Biol Phys. 2017;99:1216–1224. 29165286 10.1016/j.ijrobp.2017.07.004

[37] Antohe M Nedelcu RI Nichita L . Tumor infiltrating lymphocytes: the regulator of melanoma evolution (Review). Oncol Lett. 2019;17:4155–4161. 30944610 10.3892/ol.2019.9940 PMC6444298

[38] Hamy AS Bonsang-Kitzis H De Croze D . Interaction between molecular subtypes and stromal immune infiltration before and after treatment in breast cancer patients treated with neoadjuvant chemotherapy. Clin Cancer Res. 2019;25:6731–6741. 31515462 10.1158/1078-0432.CCR-18-3017

[39] Gooden MJM De Bock GH Leffers N . The prognostic influence of tumour–infiltrating lymphocytes in cancer: a systematic review with metaanalysis. Br J Cancer. 2011;105:93–103. 21629244 10.1038/bjc.2011.189 PMC3137407

[40] Goc J Germain C Vo-Bourgais TKD . Dendritic cells in tumor- associated tertiary lymphoid structures signal a th1 cytotoxic immune contexture and license the positive prognostic value of infiltrating CD8+ t cells. Cancer Res. 2014;74:705–715. 24366885 10.1158/0008-5472.CAN-13-1342

[41] Bergomas F Grizzi F Doni A . Tertiary intratumor lymphoid tissue in colo–rectal cancer. Cancers (Basel). 2012;4:1–10. 10.3390/cancers4010001 PMC3712686 24213222

[42] Cabrita R Lauss M Sanna A . Tertiary lymphoid structures improve immunotherapy and survival in melanoma. Nature. 2020;577:561–565. 31942071 10.1038/s41586-019-1914-8

[43] Colbeck EJ Ager A Gallimore A . Tertiary lymphoid structures in cancer: drivers of antitumor immunity, immunosuppression, or Bystander Sentinels in disease? Front Immunol. 2017;8:1–18. 29312327 10.3389/fimmu.2017.01830 PMC5742143

[44] Sautès-Fridman C Petitprez F Calderaro J . Tertiary lymphoid structures in the era of cancer immunotherapy. Nat Rev Cancer. 2019;19:307–325. 31092904 10.1038/s41568-019-0144-6

[45] Wang Y Lin HC Huang MY . The Immunoscore system predicts prognosis after liver metastasectomy in colorectal cancer liver metastases. Cancer Immunol Immunother. 2018;67:435–444. 29204700 10.1007/s00262-017-2094-8 PMC11028131

[46] Kwak Y Koh J Kim DW . Immunoscore encompassing CD3+ and CD8+ T cell densities in distant metastasis is a robust prognostic marker for advanced colorectal cancer. Oncotarget. 2016;7:81778–81790. 27835889 10.18632/oncotarget.13207 PMC5348429

[47] Galon J . Type, density, and location of immune cells within human colorectal tumors predict clinical outcome. Science. 2006;313:1960–1964. 17008531 10.1126/science.1129139

[48] Enomoto K Sho M Wakatsuki K . Prognostic importance of tumourinfiltrating memory T cells in oesophageal squamous cell carcinoma. Clin Exp Immunol. 2012;168:186–191. 22471279 10.1111/j.1365-2249.2012.04565.x PMC3390519

[49] Asano Y Kashiwagi S Goto W . Prediction of treatment response to neoadjuvant chemotherapy in breast cancer by subtype using tumor-infiltrating lymphocytes. Anticancer Res. 2018;38:2311–2321. 29599354 10.21873/anticanres.12476

[50] Khoury T Peng X Yan L . Tumor-infiltrating lymphocytes in breast cancer: Evaluating interobserver variability, heterogeneity, and fidelity of scoring core biopsies. Am J Clin Pathol. 2018;150:441–450. 30052720 10.1093/ajcp/aqy069

[51] Balermpas P Michel Y Wagenblast J . Tumour–infiltrating lymphocytes predict response to definitive chemoradiotherapy in head and neck cancer. Br J Cancer. 2014;110:501–509. 24129245 10.1038/bjc.2013.640 PMC3899751

[52] Yasuda K Nirei T Sunami E . Density of CD4(+) and CD8(+) T lymphocytes in biopsy samples can be a predictor of pathological response to chemoradiotherapy (CRT) for rectal cancer. Radiat Oncol. 2011;6:49. 21575175 10.1186/1748-717X-6-49 PMC3120676

[53] Koelzer VH Lugli A Dawson H . CD8/CD45RO T-cell infiltration in endoscopic biopsies of colorectal cancer predicts nodal metastasis and survival. J Transl Med. 2014;12. 81. 24679169 10.1186/1479-5876-12-81 PMC4022053

[54] Van den Eynde M Mlecnik B Bindea G . The link between the multiverse of immune microenvironments in metastases and the survival of colorectal cancer patients. Cancer Cell. 2018;34:1012–1026.e3. 30537506 10.1016/j.ccell.2018.11.003

[55] Yamamoto S Kato K . Immuno–oncology for esophageal cancer. Futur Oncol. 2020;16:2673–2681. 10.2217/fon-2020-0545 32777942

[56] Baba Y Nomoto D Okadome K . Tumor immune microenvironment and immune checkpoint inhibitors in esophageal squamous cell carcinoma. Cancer Sci. 2020;111:3132–3141. 32579769 10.1111/cas.14541 PMC7469863

[57] Fridman WH Pagès F Saut?s-Fridman C . The immune contexture in human tumours: impact on clinical outcome. Nat Rev Cancer. 2012;12:298–306. 22419253 10.1038/nrc3245

[58] Teng MWL Ngiow SF Ribas A . Classifying cancers basedon T-cell infiltration and PD-L1. Cancer Res. 2015;75:2139–2145. 25977340 10.1158/0008-5472.CAN-15-0255 PMC4452411

[59] Duan Q Zhang H Zheng J . Turning cold into hot: firing up the tumor microenvironment. Trends Cancer. 2020;6:605–618. 32610070 10.1016/j.trecan.2020.02.022

[60] Galon J Bruni D . Approaches to treat immune hot, altered and cold tumours with combination immunotherapies. Nat Rev Drug Discov. 2019;18:197–218. 30610226 10.1038/s41573-018-0007-y

[61] Ochoa de Olza M Navarro Rodrigo B Zimmermann S . Turning up the heat on non-immunoreactive tumours: opportunities for clinical development. Lancet Oncol. 2020;21:e419–e430. 32888471 10.1016/S1470-2045(20)30234-5

