# Supplementary material for: Immunoscore Signatures in Surgical Specimens and Tumor-Infiltrating Lymphocytes in Pretreatment Biopsy Predict Treatment Efficacy and Survival in Esophageal Cancer
Source: Ann Surg. 2021 Jul 29;277(3):e528–37. doi: 10.1097/SLA.0000000000005104 (PMC10060045; doi:10.1097/SLA.0000000000005104)
Supplement: Supplementary file 1 [file sla-277-e528-s001.docx]

**Supplimental TABLE 1.** **Uni- and multivariate analysis of pathological responders in pre-therapeutic tumor biopsy cohort (N = 146)**

| N = 146 |  | Univariate analysis | | Multivariate analysis | | | |
| --- | --- | --- | --- | --- | --- | --- | --- |
|  |  |  |  | Model A | | Model B | |
|  |  | OR (95% CI) | *P* value | OR (95% CI) | *P* value | OR (95% CI) | *P* value |
| Age (years) | >70  <70 | 1  1.32 (0.64–1.56) | 0.4481 |  |  |  |  |
| Sex | Male  Female | 1.28 (0.52–3.14)  1 | 0.5907 |  |  |  |  |
| Location | Ut  Mt / Lt | 1.08 (0.51–2.26)  1 | 0.8443 |  |  |  |  |
| Histological differentiation (SCC) | well / mod  poor | 1.67 (0.66–4.21)  1 | 0.2846 |  |  |  |  |
| NAC-regimen | DCF*  FAP** | 2.18 (1.12–4.22)  1 | **0.0210** | 2.22 (1.12–4.39)  1 | **0.0219** | 2.22 (1.14–4.35)  1 | **0.0196** |
| cT | 1, 2  3, 4 | 1  1.36 (0.63–2.92) | 0.4340 |  |  |  |  |
| cN | 0, 1  2, 3 | 1.53 (0.73–3.21)  1 | 0.2676 |  |  |  |  |
| cM | 0  1 | 2.29 (0.75–6.90)  1 | 0.1486 |  |  |  |  |
| CD3^+^ density | low: 0, 1, 2  high: 3, 4 | 1  2.58 (1.33–5.04) | **0.0053** | 1  1.75 (1.12–2.78) | **0.0169** |  |  |
| CD8^+^ density | low: 0, 1, 2  high: 3, 4 | 1  1.83 (0.95–3.54) | **0.0696** |  |  | 1  1.88 (0.96–3.68) | **0.0169** |

*DCF: docetaxel / cisplatin / 5-fluorouracil; **FAP: 5-fluorouracil / adriamycin / cisplatin
